# Structural understanding of non-nucleoside inhibition in an elongating herpesvirus polymerase

**DOI:** 10.1038/s41467-021-23312-8

**Published:** 2021-05-24

**Authors:** Robert P. Hayes, Mee Ra Heo, Mark Mason, John Reid, Christine Burlein, Kira A. Armacost, David M. Tellers, Izzat Raheem, Anthony W. Shaw, Edward Murray, Philip M. McKenna, Pravien Abeywickrema, Sujata Sharma, Stephen M. Soisson, Daniel Klein

**Affiliations:** 1 Computational and Structural Chemistry, Merck & Co., Inc., West Point, PA USA; 2 Quantitative Biosciences, Merck & Co., Inc., West Point, PA USA; 3 Discovery Chemistry, Merck & Co., Inc., West Point, PA USA; 4 Infectious Diseases and Vaccines, Merck & Co., Inc., West Point, PA USA

**Keywords:** Herpes virus, X-ray crystallography

## Abstract

All herpesviruses encode a conserved DNA polymerase that is required for viral genome replication and serves as an important therapeutic target. Currently available herpesvirus therapies include nucleoside and non-nucleoside inhibitors (NNI) that target the DNA-bound state of herpesvirus polymerase and block replication. Here we report the ternary complex crystal structure of Herpes Simplex Virus 1 DNA polymerase bound to DNA and a 4-oxo-dihydroquinoline NNI, PNU-183792 (PNU), at 3.5 Å resolution. PNU bound at the polymerase active site, displacing the template strand and inducing a conformational shift of the fingers domain into an open state. These results demonstrate that PNU inhibits replication by blocking association of dNTP and stalling the enzyme in a catalytically incompetent conformation, ultimately acting as a nucleotide competing inhibitor (NCI). Sequence conservation of the NCI binding pocket further explains broad-spectrum activity while a direct interaction between PNU and residue V823 rationalizes why mutations at this position result in loss of inhibition.

## Abstract (web summary)

Various herpesvirus therapeutics target the viral DNA polymerase. Here, the authors present the crystal structure of herpesvirus polymerase in the elongating state with bound primer-template DNA and the broad-spectrum non-nucleoside inhibitor PNU-183792, which is of interest for further drug design.

## Introduction

Human herpesviruses cause a wide spectrum of illnesses and can be classified into three subfamilies (α, β, γ)^1^. The alphaherpesvirinae include herpes simplex virus 1 (HSV1), herpes simplex virus 2 (HSV2), and varicella zoster virus (VZV). Common characteristics of this family include primary infection of mucoepithelia with latency in the sensory and cranial nerve ganglia^2^. HSV1 and HSV2 infections cause oral and genital herpes, respectively, while VZV infection is responsible for chickenpox and shingles. The betaherpesvirinae include cytomegalovirus (CMV), human herpes virus 6 (HHV6), and human herpes virus 7 (HHV7). These viruses infect various subsets of leukocytes leading to roseola in infants (HHV6/7) and mononucleosis (CMV)^2^. CMV infection can also lead to severe disease (e.g., pneumonia, retinitis, hepatitis, encephalitis) in immunocompromised patients and transplant recipients^3^. Lastly, the gammaherpesvirinae include Epstein–Barr virus (EBV) and human herpes virus 8 (HHV8) which can infect epithelial cells, lymphocytes, and establish latency in B-cells^2^. EBV is associated with mononucleosis, nasopharyngeal carcinoma, and lymphoma, while HHV8 is mainly associated with Kaposi’s sarcoma^4^. Despite this extraordinary variation in cell tropism and pathology, the herpesviruses share a common replication cycle and conserved DNA polymerase which is the target of several currently approved anti-herpetic drugs^5,6^.

Herpesvirus polymerases belong to the B-family DNA polymerases which include eukaryotic polymerases (α, δ, ε, ζ), amongst other archaeal, bacterial, bacteriophage, and viral polymerases^7,8^. A crystal structure of HSV1 polymerase (HSV1 pol, UL30) in the unliganded state has served as a representative model for the herpesvirus polymerases and revealed a conserved B-family architecture with palm, fingers, and thumb domains arranged around a central channel capable of binding primer+template duplex DNA^9^. HSV1 pol harbors 5′-3′ polymerase activity, 3′-5′ proofreading exonuclease activity, and is critical for viral genome replication. Additionally, HSV1 pol interacts with a processivity factor (UL42) and functions in the context of a larger multicomponent replisome complex—reviewed in refs. ^5,10^. The HSV1 pol apo structure coupled with a wealth of information for other B-family polymerases has enabled modeling for the herpesvirus polymerases, however no experimentally determined structures of the elongating or editing complexes have been reported.

**Fig. 1:**
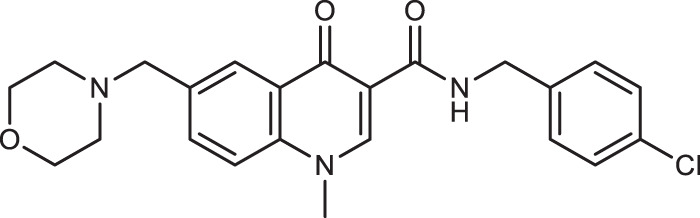
Chemical structure of PNU-183792. PNU-183792 is a non-nucleoside inhibitor of herpesvirus polymerase.

Drugs capable of inhibiting the viral polymerase include acyclic nucleoside analogs which require further phosphorylation for activity (e.g., acyclovir, gancyclovir, cidofovir, and penciclovir), and the pyrophosphate mimic foscarnet^11–13^. While viral polymerase inhibition remains an important therapeutic strategy, nucleoside inhibitors carry certain toxicity and drug-resistance liabilities, suggesting a need for further development of non-nucleoside inhibitors (NNI) of herpes polymerases. One such class of NNI, the 4-oxo-dihydroquinolines represented by PNU-183792 (PNU) (Fig. 1), was previously reported to inhibit replication for five of the eight herpesviruses known to cause disease in humans (HSV1/2, CMV, VZV, and HHV8) while maintaining selectivity against human B-family polymerases α and δ^14,15^. Additional characterization of this compound class revealed competitive inhibition of nucleotide substrate binding, but no cross-resistance with nucleotide analogs such as acyclovir-/ganciclovir-triphosphate suggesting differences in binding mode and an opportunity for treatment of nucleoside resistant strains^16^. PNU specifically binds the DNA-bound form of HSV1 pol and structural modeling suggests PNU could only be accommodated at the polymerase active site when coupled to local rearrangement or displacement of the DNA template strand^9,17^. The details of PNU binding and associated protein/DNA dynamics remain elusive, underscoring the need for DNA- and ligand-bound structural studies of herpes polymerases to further enable drug design.

To address these questions, and to develop a broader understanding of replication and editing by the herpesvirus polymerases, we determined the ternary complex crystal structure of HSV1 pol with DNA and PNU. The structure revealed an atypical binding mode and mechanism of inhibition for PNU at the polymerase active site, similar to other nucleotide competing inhibitors (NCI), that rationalizes the broad-spectrum activity and resistance mutations previously observed with this compound. Comparison of the HSV1 pol ternary complex with related B-family polymerases provides new insight into the structural elements important for DNA recognition, proofreading, and processivity across the herpesvirus polymerases.

## Results and Discussion

### Global structure of the HSV1 pol ternary complex

**Fig. 2:**
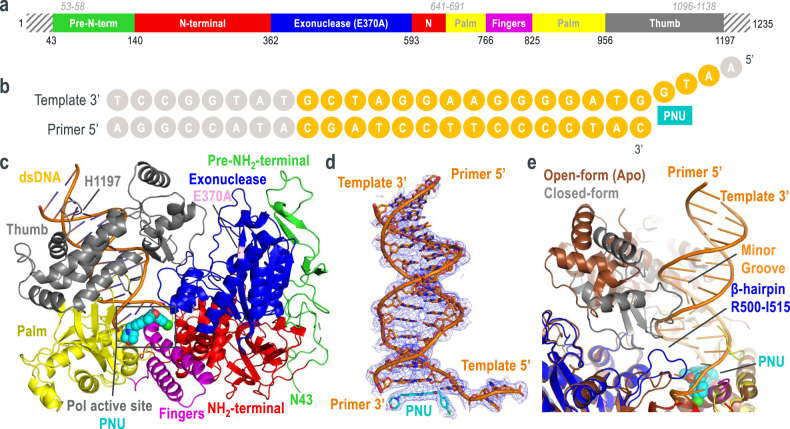
Global structure of HSV1 pol ternary complex. **a** Domain organization of HSV1 pol construct used for crystallographic studies. Residues ranges not present in this construct are shown as boxes with gray hash marks. Disordered residue ranges are labeled in gray italics. **b** Primer-template DNA schematic. DNA nucleotides are shown as circles. Gray circles represent nucleotides that were not resolved in the crystal structure. **c** HSV1 pol ternary complex structure. Protein and primer/template DNA are represented as cartoons. PNU is represented as spheres at the polymerase active site. **d** 2F_O_-F_C_ electron density (contoured at 1σ) for primer-template DNA and PNU. **e** Overlay of HSV1 pol apo and ternary complex structures highlighting thumb rotation to closed-form. The HSV1 pol apo structure is colored brown.

The HSV1 pol construct used for crystallography was nearly full-length and consisted of residues 43–1197, with N-/C- terminal truncations from residues 1–42 and 1198–1235 (Fig. 2a). An exonuclease-deficient mutation (E370A)^18^ was incorporated into this construct to prevent exonucleolytic degradation at the 3′ end of the primer strand. The resulting construct was confirmed to retain polymerase activity comparable to wild-type HSV1 pol enzyme (Supplementary Fig. 1). The primer-template DNA consisted of a 23-mer duplex and four bases of single-stranded overhang at the 5′ end of the template to mimic the elongation state (Fig. 2b). This general substrate design and sequence composition were inspired by the crystal structure of yeast pol δ^19^.

The ternary complex was assembled for crystallization by incubating purified HSV1 pol with DNA and PNU. Diffracting crystals were obtained and the structure was solved by molecular replacement at 3.5 Å resolution (Fig. 2c, d and Supplementary Table 1). The structure contained two molecules per asymmetric unit (Supplementary Fig. 2) with both molecules adopting very similar conformations (Cα RMSD = 0.232 Å). Further descriptions refer to the ternary complex associated with protein chain B due to higher electron density map quality. The HSV1 pol ternary complex adopts the common B-family architecture with the protein domains forming a partially closed channel that encircles the primer-template DNA. The N-terminal and exonuclease domains form one wall of the polymerase that guides the 5′ single-stranded template to the pol active site. The adjacent pol active site cavity is largely formed by the conserved palm and finger domains, with additional contribution from the primer-template duplex. The Fo-Fc difference map contained clear density for PNU at the pol active site cavity (Supplementary Fig. 3). The exo active site harboring the E370A mutation was positioned ~40 Å away from the pol active site. Structural overlay of HSV1 pol with yeast pol δ revealed very similar structures at the exo active site, although no metal ions were visible for HSV1 pol despite the inclusion of 5 mM MgCl_2_ during crystallization (Supplementary Fig. 4). The large distance between the HSV1 pol and exo active sites suggests the E370A mutation is unlikely to confound any analysis at the pol active site. Extending away from the pol active site, 15 bp of primer-template duplex were resolved in the central exit channel. This agrees well with DNA footprinting experiments that showed HSV1 pol can protect 14 bp of the 3′ duplex region^20^. Furthermore, the C-terminal residue H1197 resides near the end of the DNA duplex which is consistent with earlier observations that the HSV1-pol C-terminal residues 1200–1235 form a direct interaction with the processivity factor UL42^21^. Superposition of the ternary complex and apo-form structures revealed many similarities (RMSD = 1.2 Å, 786 Cα atoms), and differences were concentrated around regions involved in DNA binding (Fig. 2e). The association of the primer-template duplex was facilitated by a large conformational rotation of the thumb domain from the open- to closed-form. This brings the thumb into close contact with the primer-template duplex and packs the tip of the thumb against the minor groove. Thumb closure upon DNA binding has been observed across the B-family polymerases and the closed-form structure in HSV1 pol is very similar to that observed in the ternary complex structures of pol α, δ, ε, and ζ^19,22–24^. Differences were also noted for the exonuclease β-hairpin (residues R500-I515) which guides the single-stranded template in the ternary complex but participated in crystal packing contacts in the apo structure. Outside of the DNA binding domain, the previously unresolved pre-N-terminal region (N43-V52) was ordered at a crystal packing interface adjacent to the N-terminal domain (Fig. 2c and Supplementary Fig. 5). Residues 44–49 of this region are conserved across the human herpesvirus polymerases and earlier mutation/deletion studies in this region led to defects in viral DNA synthesis, viral yield, and establishment of latency^25,26^. It seems plausible that this region could serve as an interaction point with other replisome binding partners, however the exact structural and functional role of these residues remains unclear.

### DNA binding and recognition by HSV1 pol

**Fig. 3:**
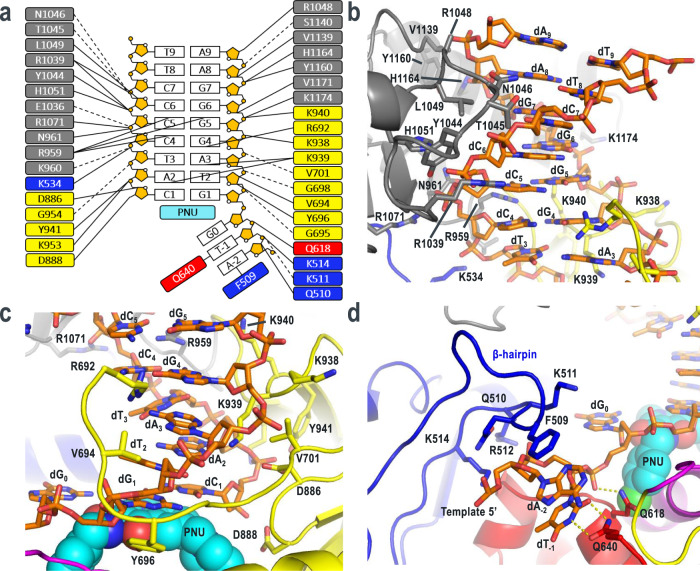
HSV1 pol—DNA contacts. **a** Schematic of protein—DNA interactions. Solid lines represent side chain interactions with DNA. Dashed lines represent main chain interactions with DNA. **b** Detailed view highlighting thumb domain interactions with the primer-template minor groove. **c** Detailed view of interactions between the palm domain and DNA. **d** Exonuclease β-hairpin and N-terminal domain interactions with the 5′ single-stranded template. Hydrogen bonds are shown as yellow dashes.

HSV1 pol forms an extensive series of contacts with the primer-template duplex (Fig. 3a). The exonuclease domain residue K534, thumb domain residues R959-N961, E1036, R1039-H1051, R1071, V1139-S1140, Y1160, H1164, V1171, K1174, and palm domain residues K938-Y941, K953-G954, R692-V701, D886, D888 encircle the duplex forming interactions predominantly with the minor groove and sugar-phosphate backbone. The most distant DNA contacts from the pol active site are formed between the thumb domain residues R1048, V1139-S1140, Y1160, H1164 and the template strand backbone at positions dA_8_ and dA_9_ (Fig. 3a, b). No contacts were visible between the thumb and dT_8_ or dT_9_ on the primer strand. Moving down the duplex to base pairs 4–7, a series of sugar-phosphate contacts continue with the thumb domain. Additionally, residues R959-N961 and R1039-H1051 pack against the minor groove. Base pairs 1–4 form interactions predominantly with the palm domain (Fig. 3c). Palm residues K938–940 pack against the minor groove and residues Y941, K953-G954, D886, D888 form interactions with the primer strand backbone. The loop formed by residues R692-V701 wraps around the template strand from the minor groove and enters the major groove, forming an interaction between the sidechain of R692 and the nucleobase of dG4. Electron density from residues 641–691 was untraceable, however this region is not conserved across the herpesvirus polymerases suggesting it may play a less important role for function. Residues K939 and R959 were especially noteworthy as they formed interactions with the minor groove edges of base pairs 2–3 and 4–5, respectively. These interactions are reminiscent of the DNA binding and minor groove “sensing” contacts in yeast pol δ in which residues form direct or water-mediated hydrogen bonds with the universal pyrimidine-O2/purine-N3 atoms to test base pair geometry up to 5 bp from the pol active site^19^. Structural superposition and sequence alignment of HSV1 pol with yeast pol δ revealed similar global structures (RMSD = 2.6 Å) and local conservation of these important DNA contacts, despite only 19% sequence identity overall. (Supplementary Fig. 6a, b). Furthermore, these residues are largely conserved across all human herpesvirus polymerases suggesting a conserved mechanism of mismatch detection, distant from the pol active site, that may serve as a trigger for shuttling of the primer strand to the exo active site for editing (Supplementary Fig. 6c).

The 5′ single-stranded region of the template DNA strand in our construct represents part of the DNA replication bubble and threads into the polymerase active site though a channel formed by the β-hairpin residues R500-I515 of the exonuclease domain and the tip of α-helix 618–632 of the N-terminal domain (Fig. 3d). Residues Q618 and Q640 form hydrogen bonds with the phosphate of dG_0_ and nucleobase of dT_−1_, respectively. The β-hairpin residues Q510, K511, and K514 also interact with the DNA backbone at positions dA_−2_ and dT_−1_, while F509 forms a stacking interaction with the dA_−2_ nucleobase. The β-hairpin element is present in other B-family polymerases and has been shown to play a role in maintaining DNA association to pol during mismatch excision and stabilizing the melted duplex at the exo site^27,28^. The β-hairpin sequence is quite divergent for the herpesvirus polymerases; however, the identified DNA binding residues are largely conserved suggesting this element may interact similarly with the 5′ template strand across the family (Supplementary Fig. 7).

### PNU-183792 adopts an atypical binding mode at the HSV1 pol active site

**Fig. 4:**
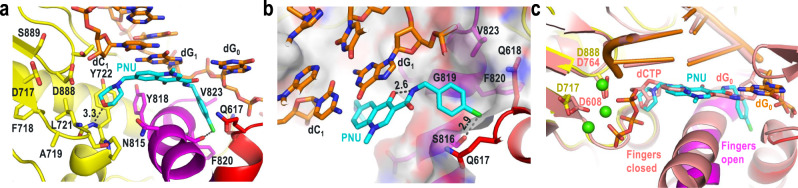
HSV1 polymerase active site and interactions with PNU-183792. **a** HSV1 polymerase active site and interactions with PNU. **b** PNU p-chlorobenzyl interactions and intramolecular hydrogen bonding. **c** Overlay of HSV1 pol and yeast pol δ (PDB:3IAY [10.2210/pdb3iay/pdb]) ternary complex structures at polymerase active site. The yeast pol δ protein and active site Ca^2+^ ions are colored salmon and green, respectively.

The PNU molecule fully occupied the polymerase active site cleft, overlapping with the binding site of incoming dNTPs and forming interactions with the surrounding protein and DNA (Fig. 4a and Supplementary Fig. 8). The central methylquinolinone of PNU forms stacking interactions with the terminal base pair of the growing DNA duplex (dC_1_:dG_1_). This base pair forms one side of the drug binding pocket. The morpholine group occupies the space that normally accepts an incoming dNTP during the elongation cycle and is anchored by a hydrogen bond between the morpholine oxygen and backbone amide of Y722. No metal ions were observed at the catalytic site aspartate residues D717 and D888, despite the inclusion of MgCl_2_ during crystallization. This is not unexpected as there is no incoming triphosphate group or metal chelating group in PNU. Furthermore, PNU would not be expected to participate in any interactions with the catalytic metals as the morpholine group is quite distant from the primer 3′ hydroxyl, which resides ~5 Å away. Interestingly, it was previously reported that the affinity of PNU for the HSV1 pol-DNA duplex was increased in the presence of Mg^2+^ or Ca^2+^ ions^9^. In the absence of a direct PNU-metal interaction, another possible explanation for the increased potency could be further stabilization of the DNA bound state. At the opposite end of PNU, the p-chlorobenzyl group displaced the template base dG_0_ from the position required to base pair with incoming dNTP. The extensive interactions observed between PNU, the dC_1_:dG_1_ base pair, and the dG_0_ template base rationalizes earlier observations that PNU selectively binds the elongating state of HSV1 pol. Moreover, these interactions do not appear DNA sequence dependent, suggesting that PNU should inhibit HSV1 pol at any point in the elongation stage of viral replication. The p-chlorobenzyl binding pocket is formed by the hydrophobic moieties of residues Q617, Q618, G819, F820, and V823, with one polar contact between the S816 side chain hydroxyl and para-chlorine atom of PNU (Fig. 4b and Supplementary Fig. 8). An intramolecular hydrogen bond was also noted between the amide nitrogen and methylquinolinone oxygen atoms of PNU which appears to stabilize its bioactive conformation. This binding mode is consistent with earlier predictive modeling^9^ and the fact that V823A was shown to cause resistance to PNU^14^. PNU inhibition was hindered in vitro either when the naturally occurring valine in HSV1, CMV, or HHV8 was mutated to alanine (4- to 10-fold IC_50_ increase), or in HHV6 and pol δ which naturally contain alanine at this position. Interestingly, back mutation to valine in HHV6 resulted in a 4–5 fold improvement in the PNU IC_50_, although the same change had no effect on the pol δ IC_50_^14^. Taken together, these data provide strong evidence for V823 acting as a determinant of selectivity for the 4-oxo-dihydroquinolines although the structural implications of this mutation may be subtle. Mutation from valine to alanine at this position would disrupt the Van der Waals interactions with the p-chlorobenzyl group and potentially cause differences in the positioning of template strand base which is critical for forming part of the PNU binding pocket.

Structural alignment of HSV1 pol with the yeast pol δ elongation state structure highlights the mechanism of action for PNU (RMSD = 2.6 Å) (Fig. 4c). The morpholine oxygen mimics the 3′-OH of dNTP and forms a similar hydrogen bond with the Y722 backbone, ultimately blocking the association of dNTP to the polymerase. Additionally, PNU disrupts the active site by fully displacing the template base and locking the two helices of the fingers domain into a catalytically incompetent open position. This general mechanism of inhibition is analogous to that previously observed for the NCI INDOPY-1 bound to HIV reverse transcriptase^29^ (Supplementary Fig. 9), and for the nonselective inhibitor aphidicolin bound to human pol α^22^ (Supplementary Fig. 10). PNU, INDOPY-1, and aphidicolin all occupy the polymerase active site, form stacking or hydrophobic interactions with the terminal base pair, displace the template base, lock the fingers in to the open conformation, and form hydrogen bonds with the amide backbone of the conserved tyrosine corresponding to Y722. Considering these similarities, PNU and aphidicolin would perhaps be best characterized as members of the NCI family. One notable structural difference between PNU and aphidicolin was the extension of the p-chlorobenzyl group of PNU towards a pocket with lower sequence conservation. In contrast, aphidicolin only occupies the highly conserved region of the pol active site (Supplementary Fig. 10). These unique p-chlorobenzyl contacts rationalize the superior selectivity of PNU relative to aphidicolin for the B-family polymerases. While the inhibitors described above all rely on trapping the fingers in the open state for inhibition, the non-nucleoside polymerase inhibitor foscarnet has been shown to act as a pyrophosphate mimic that can lock the fingers domain in the closed state^13,30,31^. Clinically relevant HSV1 pol mutations associated with foscarnet resistance include R700G, A719V, and S724N^5,32–34^. The proximity of the palm residues A719 and S724 to the catalytic site (Fig. 4a) suggests the associated mutations could directly perturb forcarnet binding and coordination to metal, whereas the mutation R700G is more difficult to rationalize as it resides adjacent to template dA_3_, ~15 Å from the expected foscarnet binding site^13,30^. The mutation E771Q has been reported to increase HSV1 pol susceptibility to foscarnet^35^. In this structure, the residue E771 forms a salt bridge with R779 from the fingers domain, stabilizing the open conformation. Mutation at this position from E to Q would remove this stabilizing interaction and shift equilibrium towards the closed state, ultimately favoring foscarnet inhibition.

The ternary complex structure helps define the pharmacophore for PNU and provides an opportunity for structure-based drug design of novel NCIs. Little additional room is available in the p-chlorobenzyl binding pocket and only small changes are expected to be tolerated at this position. In contrast, the stacking interaction between the central methylquinolinone core and terminal base pair suggests a variety of nucleobase-like ring systems could be substituted here. Nonplanar groups may also be tolerated in this cleft as the fingers are locked in an open conformation, similar to what was observed in the pol α ternary complex with aphidicolin^22^. Only one hydrogen bond was observed between the morpholine ring and the nucleotide binding region. Optimized ring replacements in this conserved pocket may improve potency while maintaining broad-spectrum activity. Designs that mimic the observed intramolecular hydrogen bond may also help to further stabilize the bioactive conformation. Beyond structure-based drug design, this work provides a foundation for the development of improved structural systems to probe remaining questions around the details of nucleoside inhibition, active site switching, processivity, and the assembly of the larger replisome.

## Methods

### Preparation of PNU-183792

A step-by-step protocol describing the synthesis of PNU-183792 is provided in the supplementary information.

### Protein expression and purification

The HSV1-pol construct consisting of residues 43–1197 (E370A) with an N-terminal 8X HIS-SUMO-Precission fusion protein was synthesized as pBAC1 plasmid (Supplementary Table 2). Protein was expressed in *Sf*21 cells by baculoviral expression. *Sf*21 cells were cultured in 10 L of SF900 II media supplemented with 5 mg/mL gentamicin, in a 20-L wavebag up to an infection density of 6 × 10^5^ cells/mL. The cells were subsequently infected by adding 1 mL of BIIC (Baculovirus Infected Insect Cell) expressing the protein to a final MOI of 0.2 (Assuming 100 pfu/cell) at 27 °C. Cell pellets were harvested 96 h post-infection by centrifugation. The pellet was resuspended in lysis buffer consisting of 25 mM Tris pH 7.8, 150 mM NaCl, 5 mM β-mercaptoethanol, 10% glycerol, 20 mM imidazole, Roche complete protease inhibitor tablets w/o EDTA (1 tablet/50 mL buffer), and Benzonase (100 U/mL). Lysis buffer was added at a ratio of 5 mL buffer per g of pellet. Cells were disrupted by microfluidizer at 14,000 psi and the lysate was subsequently cleared by centrifugation at 38,000x*g* for 1 h at 4 °C. The supernatant was loaded to a 2 × 5 mL His-Trap column at 3 mL/min using Akta Pure. Following a 17-column volume (CV) wash at 4 mL/min, the protein was eluted with a linear gradient from 0–100% His elution buffer (25 mM Tris pH 7.8, 150 mM NaCl, 5 mM β-mercaptoethanol, 10% glycerol, 500 mM imidazole) over 15 CVs. Fractions were analyzed by SDS-PAGE, pooled, and diluted 1:1 with buffer consisting of 25 mM Tris pH 7.6, 10% w/v glycerol, 1 mM TCEP. SUMOstar Protease was added (50 U protease/1 mg protein) and incubated for 30 h at 4 °C. The resulting digest was loaded to a 2 × 5 mL HiTrap Q HP column at 1.5 mL/min and washed with 17 CVs of buffer A (25 mM Tris-HCl pH 7.6, 1 mM TCEP, 10% glycerol, 90 mM NaCl) at 3 mL/min. Protein was eluted with a gradient from 0–50% buffer B (buffer A supplemented with 600 mM NaCl) over 33 CVs. Fractions containing HSV1-pol were pooled, concentrated in an Amicon Ultra-15 filter (50,000 MWCO), and loaded to a size exclusion column (Superdex 200 16/600) equilibrated in 25 mM Tris pH 8.0, 150 mM NaCl, and 1 mM TCEP. A single monomeric peak of HSV1-pol was observed. SDS-PAGE and Bradford assay suggests HSV1-pol purity of >95% and protein concentration ~4 mg/mL. This purification scheme left an unprocessed Precission cleavage sequence (LEVLFQG) at the N-terminal of HSV1 pol.

### HSV1 pol AlphaLISA nucleotide incorporation assay

His-Trap purified HSV1-pol was utilized for the nucleotide incorporation assay. Heterodimeric nucleic acid substrate used in the herpesvirus polymerase reactions was generated by annealing a 57-mer template (5′-GAGGTCAAAACAGCGTGGATGGCGTCTCCAGGCGATCTGACGGTTCACTAAACGAGC-3′) to a 17-mer digoxigenin-labeled primer (5′-DIG-AGCTCGTTTAGTGAACC-3′) (Supplementary Table 3). Standard assay buffer of 10 mM HEPES, pH 7.5, 25 mM KCl, 12.5 mM NaCl, 5 mM MgCl_2_, 2.5% glycerol, 0.34 mg/ml bovine serum albumin, and 1 mM tris (2-carboxyethyl) phosphine was also tested with titrated imidazole to control for purification sample interference. The polymerization reactions with titrated polymerase were initiated by the addition of template/primer substrate (final concentration: 1.6 nM) and dNTPs (final concentration: 24 nM dCTP, 24 nM dGTP, 16 nM dATP, 16 nM dTTP, and 0.8 nM biotin-dUTP). After 60 min incubation at 37 °C, reactions were terminated with quench buffer (25 mM HEPES pH 7.5, 100 mM NaCl, 0.25% Tween-20, 12 mM EDTA, and 1 mg/ml bovine serum albumin). Incorporation of biotinylated UTP was detected with 5ug/ml anti-DIG AlphaLISA acceptor beads and 10 ug/ml streptavidin AlphaLISA donor beads (PerkinElmer).

### Complexation and Crystallization of the HSV1-pol ternary complex

Primer strand (5′-AGGCCATACGATCCTTCCCCTAC-3′) and template strand (5′- AATGGTAGGGGAAGGATCGTATGGCCT-3′) oligos were obtained from Millipore Sigma (Supplementary Table 3). Oligos were resuspended in annealing buffer (10 mM Tris pH 7.6, 50 mM NaCl), and annealed to form the duplex. Purified HSV1-pol (4 mg/mL) was mixed with primer-template duplex (1:1 molar ratio) in complexation buffer (25 mM Tris-HCl pH 8.0, 150 mM NaCl, 1 mM TCEP, and 5 mM MgCl_2_), and incubated on ice for 30 min. PNU-183792 was then added from a 100 mM dimethyl sulfoxide stock to a final concentration of 50 uM and incubated on ice for 30 min. The resulting ternary complex was concentrated in an Amicon Ultra (100 kDa MWCO) filter to ~40 mg/mL. Crystals were grown at 10 C by hanging drop vapor diffusion over reservoir solution containing 0.1 M Na HEPES pH 6.8 and 0.4–0.7 M disodium tartrate. Ternary complex and reservoir solution were mixed at 1:1 or 1:2 ratio and crystals appeared after 2–3 days, with continued growth for 2 weeks. Crystals were harvested into motherliquor supplemented with 20% (vol/vol) ethylene glycol and flash frozen in liquid nitrogen.

### Data collection and structure determination

Diffraction data were collected at the Advanced Photon Source on the Industrial Macromolecular Crystallography Association—Collaborative Access Team beamline (17-ID). Diffraction data were processed using autoPROC^36^. Phases were obtained by molecular replacement using Phaser^37^ with the apo HSV1-polymerase structure as a search model (PDB:2GV9 [10.2210/pdb2gv9/pdb]). A solution with 2 molecules/asymmetric unit was found and subsequent refinement illustrated clear density for dsDNA/PNU-183792 in the difference Fourier maps. Iterative rounds of model building and refinement were conducted in Coot^38^ and Phenix^39^. A summary of diffraction and refinement statistics can be found in Supplementary Table 1.

## Supplementary information

Supplementary Information

## Data Availability

Atomic coordinates and structure factors have been deposited in the RCSB Protein Data Bank under PDB:7LUF [10.2210/pdb7LUF/pdb]. Source data are provided with this paper. Other data are available from the corresponding author upon reasonable request. Publicly available structure data used in this study are available at Protein Data Bank under accession codes 2GV9 [10.2210/pdb2gv9/pdb], 3IAY [10.2210/pdb3iay/pdb], 4Q5V [10.2210/pdb4q5v/pdb], and 6O9E [10.2210/pdb6O9E/pdb]. Sequences used in alignments are available from Uniprot under accession numbers P04293, P89453, Q0Q8S1, Q1HVC1, P08546, P28857, Q9QJ32, P52342, Q2HRD0, and P15436. Source data are provided with this paper.

## Source data

Source Data
