# Supplementary material for: Structural understanding of non-nucleoside inhibition in an elongating herpesvirus polymerase
Source: Nat Commun. 2021 May 24;12:3040. doi: 10.1038/s41467-021-23312-8 (PMC8144222; doi:10.1038/s41467-021-23312-8)
Supplement: Supplementary file 1 — Supplementary Information [file 41467_2021_23312_MOESM1_ESM.pdf]

Supplementary Information

Synthesis of PNU-183792

Step 1: Synthesis of *N*-methyl-4-(morpholinomethyl)aniline

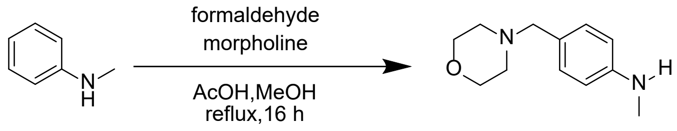

To a solution of *N*-methylaniline (20 g, 187 mmol) in MeOH (400 mL) and Acetic Acid (20 mL) was added morpholine (19.51 mL, 224 mmol) and formaldehyde (27.8 mL, 373 mmol) at room temperature. The resulting solution was stirred at reflux for 16 h. The solution was diluted with water, extracted with CH_2Cl_2 (3x800 mL), washed with water (2x500ml) and brine (500 mL), and dried over sodium sulfate. The solvent was distilled off under vacuum at 35 °C to provide crude product, which was purified by gradient elution on SiO_2 (0% to 5% MeOH in CH_2Cl_2 to afford *N*-methyl-4-(morpholinomethyl)aniline (7 g, 14.86 mmol, 7.96 % yield). LRMS (M+H) = 207.2.

Step 2: Synthesis of diethyl 2-((methyl(4-(morpholinomethyl)phenyl)amino)methylene)malonate

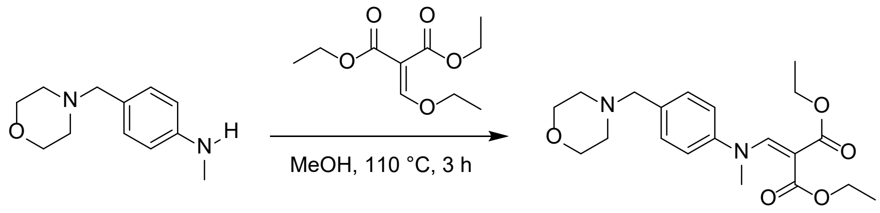

To a stirred solution of *N*-methyl-4-(morpholinomethyl)aniline (4 g, 19.39 mmol) in MeOH (20 mL) was added diethyl 2-(ethoxymethylene)malonate (4.19 g, 19.39 mmol). The solution was heated slowly to 110 °C with atmospheric distillation of the ethanol by-product. Distillation was continued for 3 h at 110 °C, and then the solution was cooled to room temperature and the residual ethanol was removed by vacuum distillation and toluene azeotrope. Further drying under high vacuum afforded diethyl 2-((methyl(4-(morpholinomethyl)phenyl)amino)-methylene)malonate (4.8g, 4.82 mmol, 24.86 % yield), which was used in the subsequent step without further purification. LRMS (M+H) = 377.2.

Step 3: Synthesis of ethyl 1-methyl-6-(morpholinomethyl)-4-oxo-1,4-dihydroquinoline-3-carboxylate

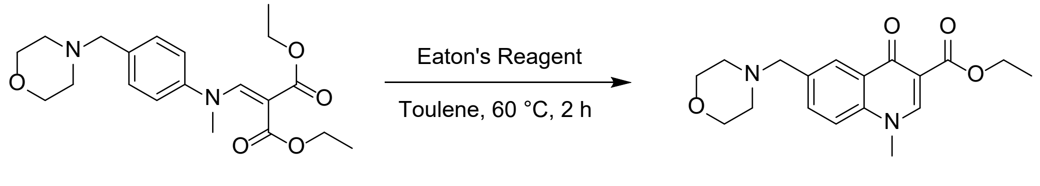

To a stirred solution of Eaton's Reagent (6.07 g, 25.5 mmol) in round-bottom flask at room temperature was added a solution of diethyl 2-((methyl(4-(morpholinomethyl)phenyl)amino)methylene)malonate (4.8

g, 12.75 mmol) in Toluene (80 mL). The solution was heated slowly to 60 °C for 2 h. The solution was cooled to ~0 °C and water (10 mL) was added. The pH of the resulting slurry was adjusted to 9.5-10.5 by the addition of 10 M NaOH (aq). The solution was diluted with water, extracted with CH_2Cl_2 (3x350mL), washed with water (2x300mL) and brine (300mL) and dried over sodium sulfate. The solvent was distilled under vacuum at 35 °C to provide crude ethyl 1-methyl-6-(morpholinomethyl)-4-oxo-1,4-dihydroquinoline-3-carboxylate (3.5 g, 7.51 mmol, 58.9 % yield), which was used in the subsequent step without further purification. LRMS (M+H) = 331.2.

Step 4: Synthesis of 1-methyl-6-(morpholinomethyl)-4-oxo-1,4-dihydroquinoline-3-carboxylic acid

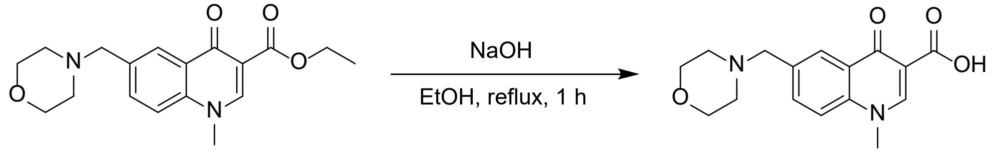

To a stirred solution of ethyl 1-methyl-6-(morpholinomethyl)-4-oxo-1,4-dihydroquinoline-3-carboxylate (180 mg, 0.545 mmol) in EtOH (5 mL) and water (5 mL) was added 50% NaOH (aq) (87 mg, 1.090 mmol) at room temperature. The reaction was heated to 90 °C for 1 h. The ethanol was removed by vacuum distillation and the aqueous layer was extracted with diethyl ether (15 mL). The pH of the aqueous layer was adjusted to 3.8–4.3 with 6 M HCl (aq) at 0~ °C. The solids were filtered, and washed with cooled water (7 mL) and diethyl ether (7 mL) to yield 1-methyl-6-(morpholinomethyl)-4-oxo-1,4-dihydroquinoline-3-carboxylic acid (60 mg, 0.155 mmol, 28.5% yield). LRMS (M+H) = 303.2.

Step 5: Synthesis of PNU-183792 (*N*-(4-chlorobenzyl)-1-methyl-6-(morpholinomethyl)-4-oxo-1,4-dihydroquinoline-3-carboxamide)

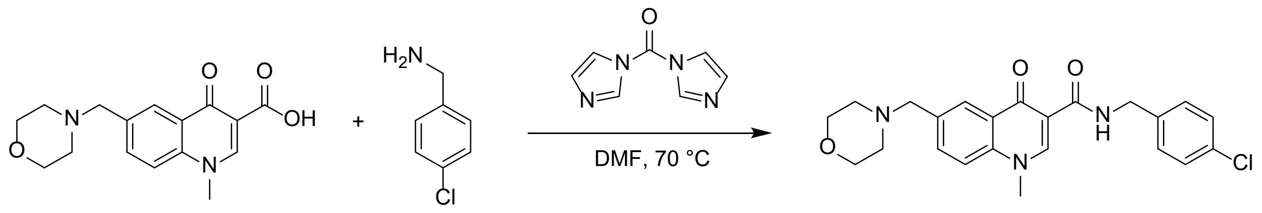

PNU-183792

To a stirred solution of 1-methyl-6-(morpholinomethyl)-4-oxo-1,4-dihydroquinoline-3-carboxylic acid (80 mg, 0.265 mmol) in DMF (500 µL) was added CDI (86 mg, 0.529 mmol), and the reaction was heated at 70 °C. After 1.5 h additional CDI (86 mg, 0.529 mmol) was added and the heating was continued. After 1 h, (4-chlorophenyl)methanamine (64.4 µl, 0.529 mmol) was added and heating was continued at 70 °C for 0.5 h. The reaction was diluted with EtOAc and water, the organic phase separated and washed with water (2x), brine, and then and dried over sodium sulfate, filtered, and concentrated *in vacuo*. The resulting residue was purified by on SiO_2 using an RF Isco system (0% to 7% MeOH/CH_2Cl_2, 12-g gold, 15 min @ 30 mL/min) to afford *N*-(4-chlorobenzyl)-1-methyl-6-(morpholinomethyl)-4-oxo-1,4-dihydroquinoline-3-carboxamide (108 mg, 96% yield). LRMS (M+H) = 426.3. ^{1}H NMR (500 MHz, DMSO-d_6) δ 10.42 (s, 1H), 8.87 (s, 1H), 8.25 (s, 1H), 7.81 (s, 2H), 7.44–7.33 (m, 4H), 4.56 (d, J = 5.8 Hz, 2H), 4.02 (s, 3H), 3.63 (s, 2H), 3.58 (s, 4H), 2.38 (s, 4H).

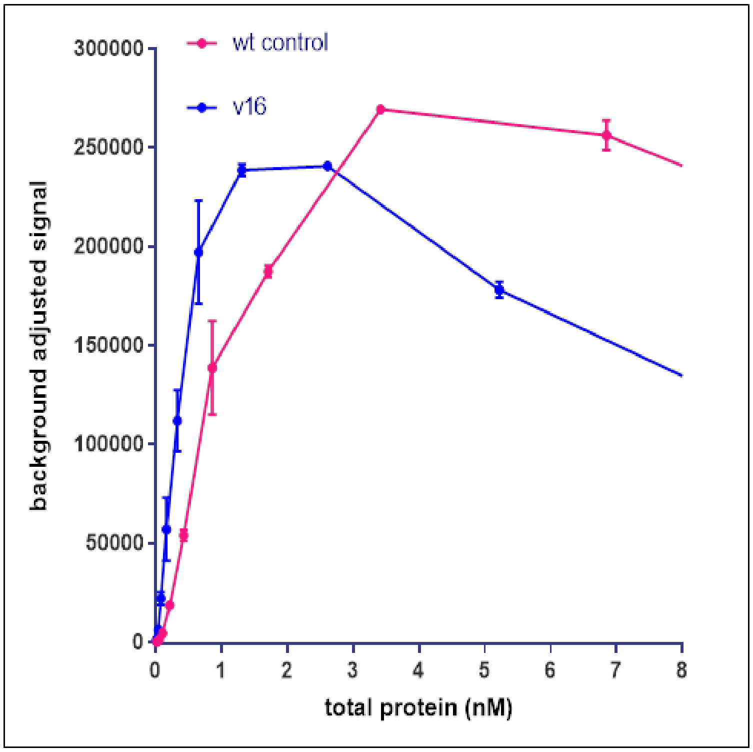

Supplementary Figure 1. The AlphaLISA nucleotide incorporation assay demonstrated comparable polymerase activity between wild-type HSV1 pol (wt control, pink line) and the HSV1 pol crystallography construct described in this work (v16, blue line). Data are presented as mean values of n=3 independent experiments and error bars represent ± standard deviation.

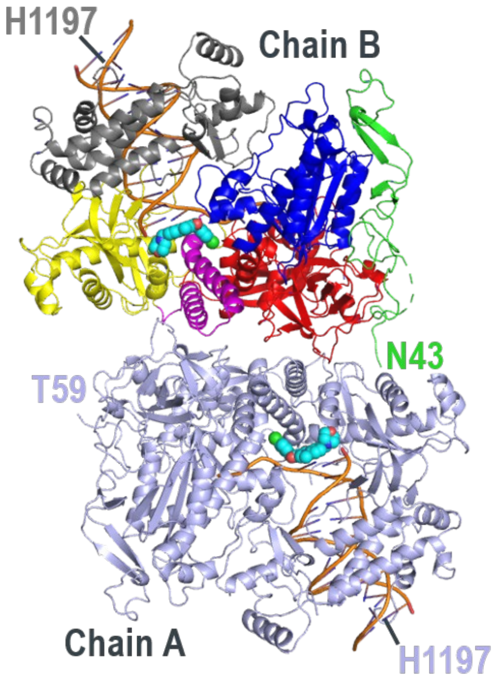

Supplementary Figure 2. HSV1 pol crystallographic dimer. The structure contained two molecules per asymmetric unit. Electron density was traced for chain A from residues 59-1197 and for chain B from 43-1197, although both molecules contained disordered regions at 53-58 in the pre-N-terminal domain, 641-691 at the N-terminal/palm domain transition, and 1096-1138 in the thumb domain. Both chains adopted similar conformations (Cα R.M.S.D. = 0.232 Å). The primer/template DNA and PNU-183792 were resolved in the electron density of both molecules, with PNU-183792 residing at the polymerase active site.

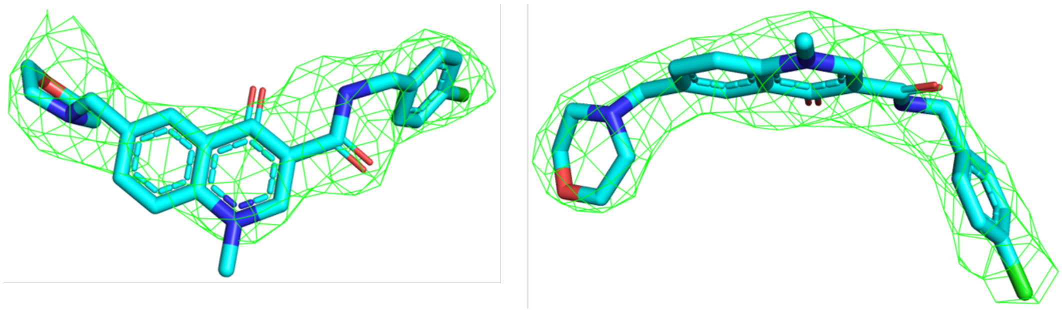

Supplementary Figure 3. Two different views of the Fo-Fc omit map (green mesh, 3.5 σ) for the region around PNU-183792.

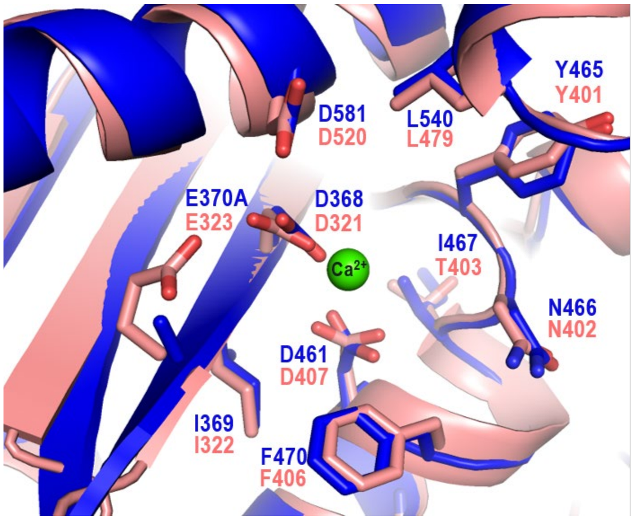

Supplementary Figure 4. Overlay of the HSV1 pol and yeast pol δ (PDB: 3IAY [http://dx.doi.org/10.2210/pdb3iay/pdb]) exonuclease active sites. HSV1 pol is colored blue. The yeast pol δ protein and Ca^{2+} ion are colored salmon and green, respectively.

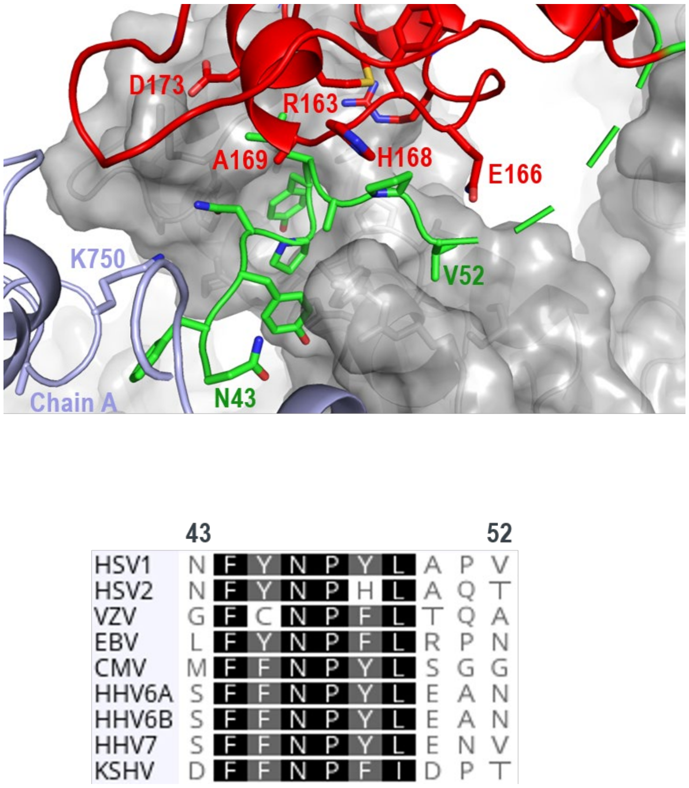

Supplementary Figure 5. Structure and conservation of pre-N-terminal residues 43-52. Residues 43-52 were ordered at an interface formed by the N-terminal domain of Chain B (red), the palm domain of Chain A (light blue), and the N-terminal domain of a symmetry related molecule (grey surface). Residues 44-49 are largely conserved across all herpesvirus polymerases. Uniprot names/accession numbers for aligned sequences from top to bottom are as follows: P04293|DPOL_HHV11 [https://www.uniprot.org/uniprot/P04293], P89453|DPOL_HHV2H [https://www.uniprot.org/uniprot/P89453], Q0Q8S1|Q0Q8S1_HHV3 [https://www.uniprot.org/uniprot/Q0Q8S1], Q1HVC1|DPOL_EBVA8 [https://www.uniprot.org/uniprot/Q1HVC1], P08546|DPOL_HCMVA [https://www.uniprot.org/uniprot/P08546], P28857|DPOL_HHV6U [https://www.uniprot.org/uniprot/P28857], Q9QJ32|DPOL_HHV6Z [https://www.uniprot.org/uniprot/Q9QJ32], P52342|DPOL_HHV7J [https://www.uniprot.org/uniprot/P52342], Q2HRD0|DPOL_HHV8P [https://www.uniprot.org/uniprot/Q2HRD0].

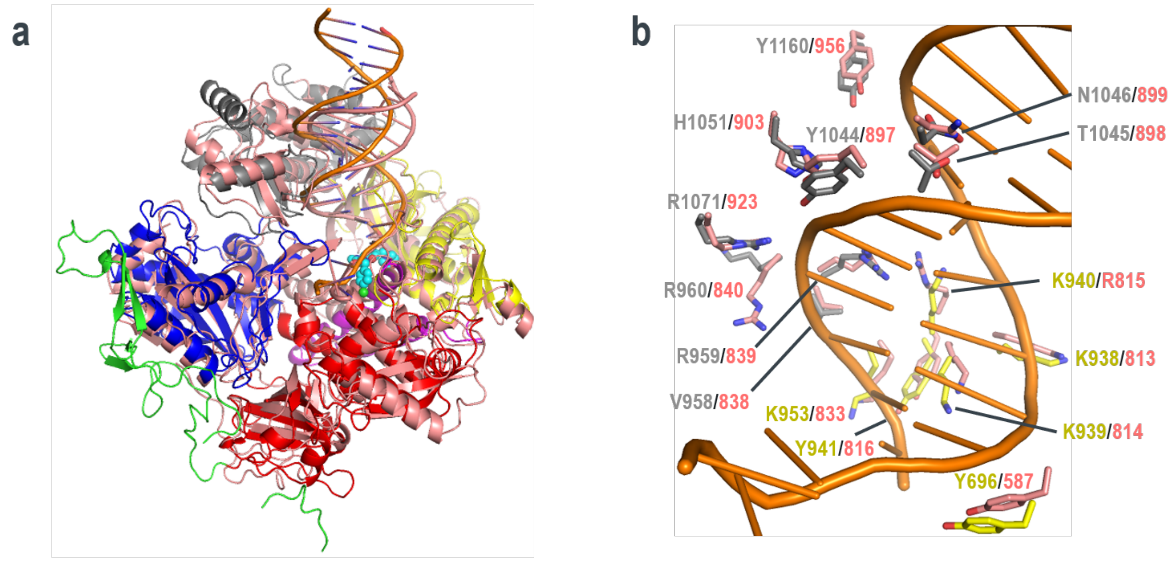

c

	692-701	938-941	953	958-961	1039-1051	1071	1160	1164	1171	1174
**HSV1**	RHVGYQGARV	KKKY	K	VRKN	RHPRAYTNKR-LAH	R	Y	H	V	K
**HSV2**	RHVGYQGARV	KKKY	K	VRKN	RHPRAYTNKR-LAH	R	Y	H	V	K
**VZV**	RNVGYKGARV	KKKY	K	VRKN	RPPSAYINRR-LAH	R	Y	H	V	K
**EBV**	DRDGYQGATV	KKRY	K	VRKT	RKLSAYKSTQ-MPH	R	H	K	N	Q
**CMV**	AAVSYQGATV	KKRY	K	VRKT	KDISLYRQSN-LPH	R	K	Q	N	S
**HHV6A**	QGIGYKGATV	KKRY	K	VRKT	KEMAAYKQPN-LAH	R	K	Q	N	S
**HHV6B**	QGIGYKGATV	KKRY	K	VRKT	KEVAAYKQPN-LAH	R	K	Q	N	S
**HHV7**	ENVGYKGATV	KKRY	K	VRKT	KDISAYKQAN-LPH	R	K	Q	N	S
**KSHV**	AVSGYQGATV	KKRY	K	IRKT	RPLADYKTQN-LPH	R	L	K	N	Q
**yPOLD**	SDDQYEGATV	KKRY	K	VRRD	---PNYTNPQ--PH	R	Y	Q	S	A

Supplementary Figure 6. Structural comparison of HSV1-pol and yeast pol δ (PDB: 3IAY [http://dx.doi.org/10.2210/pdb3iay/pdb]). (a) Secondary structure superposition of HSV1-pol with yeast pol δ (salmon color cartoon), R.M.S.D. = 2.6 Å. (b) DNA binding contacts. HSV1-pol ternary complex is represented as grey side chain sticks/orange DNA cartoon. Yeast pol δ is represented as salmon side chain sticks. (c) Sequence alignment of HSV1 pol DNA binding residues with human herpesvirus polymerases and yeast pol δ. Uniprot accession number/name for yPOLD is P15436|DPOD_YEAST [https://www.uniprot.org/uniprot/P15436]. Herpesvirus sequence information is listed in Supplementary Figure 5.

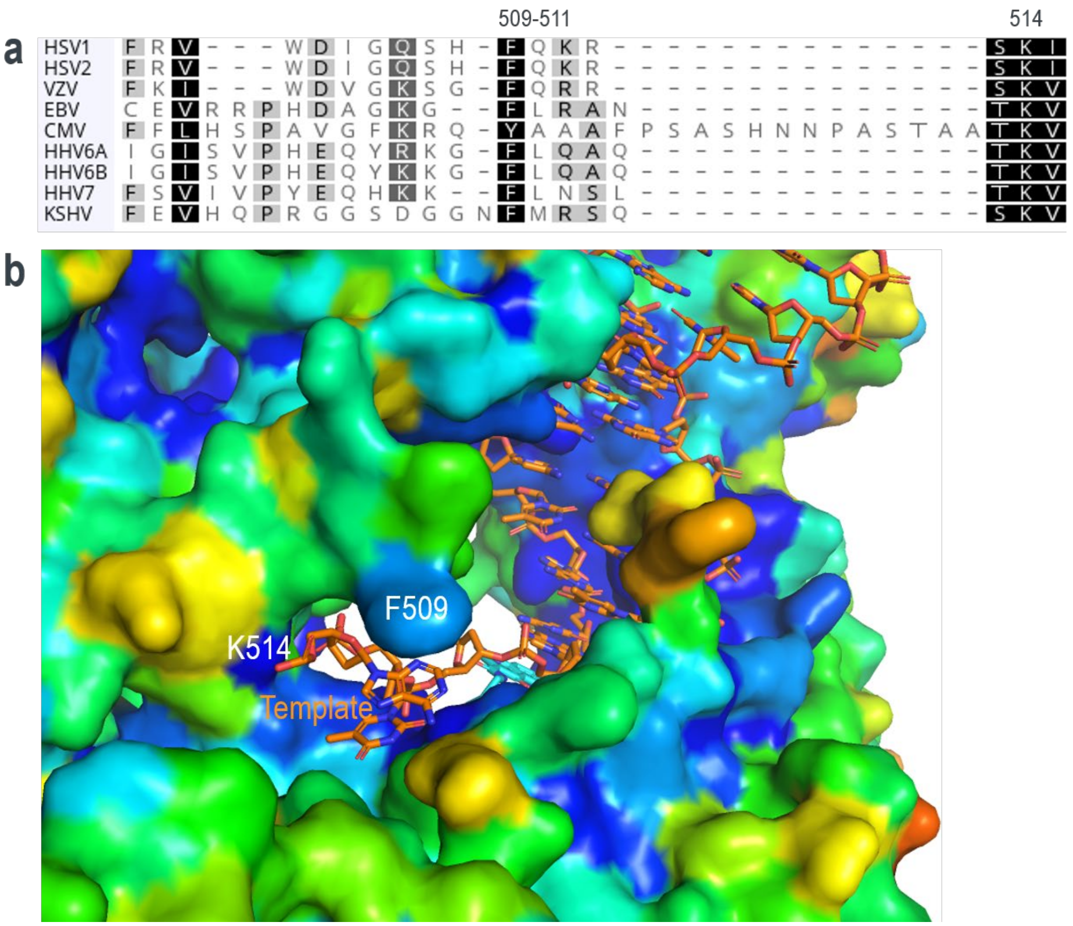

Supplementary Figure 7. Sequence conservation at the single-stranded template binding surface. (a) Sequence alignment of exonuclease β-hairpin residues 500-515. Numbering corresponds to HSV1 pol. (b) Conservation mapping of the human herpesvirus sequences onto the surface of HSV1 pol. Regions colored blue represent areas of higher conservation.

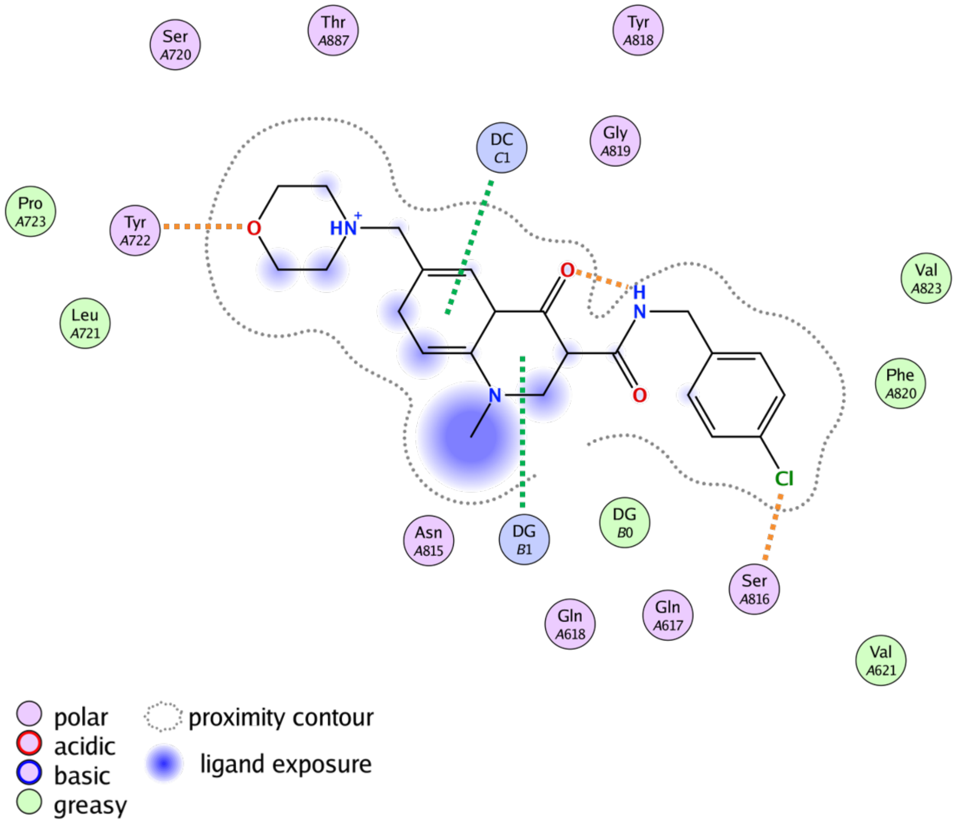

Supplementary Figure 8. PNU-183792 ligand interaction plot. Key hydrogen bonding and stacking interactions are shown as orange and green dashes, respectively.

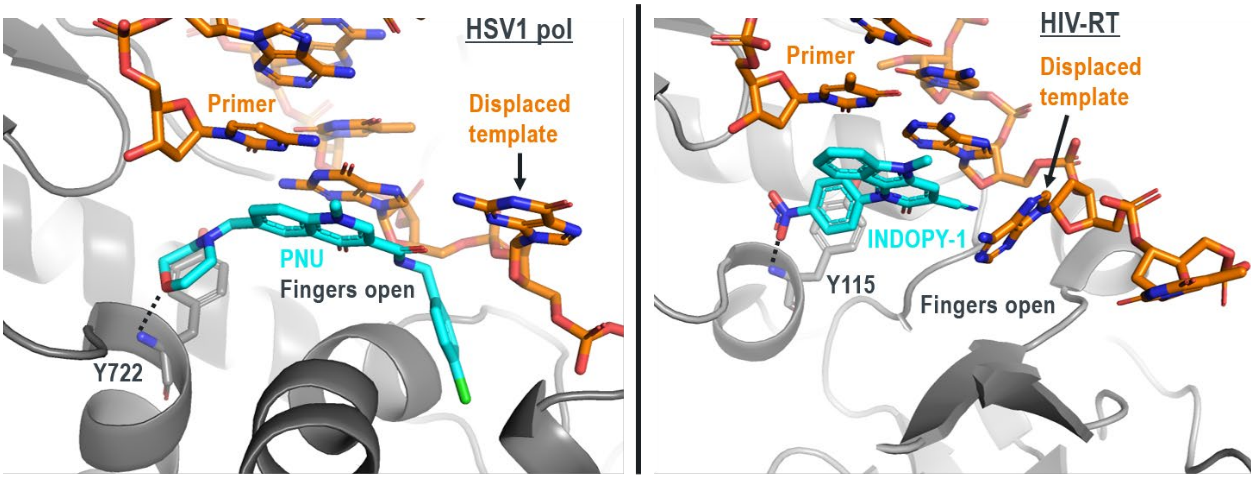

Supplementary Figure 9. PNU and INDOPY-1 adopt analogous binding modes. HSV1 pol ternary complex is shown on the left panel. The HIV reverse transcriptase ternary complex with INDOPY-1 is shown on the right panel (PDB:6O9E [http://dx.doi.org/10.2210/pdb6o9e/pdb]).

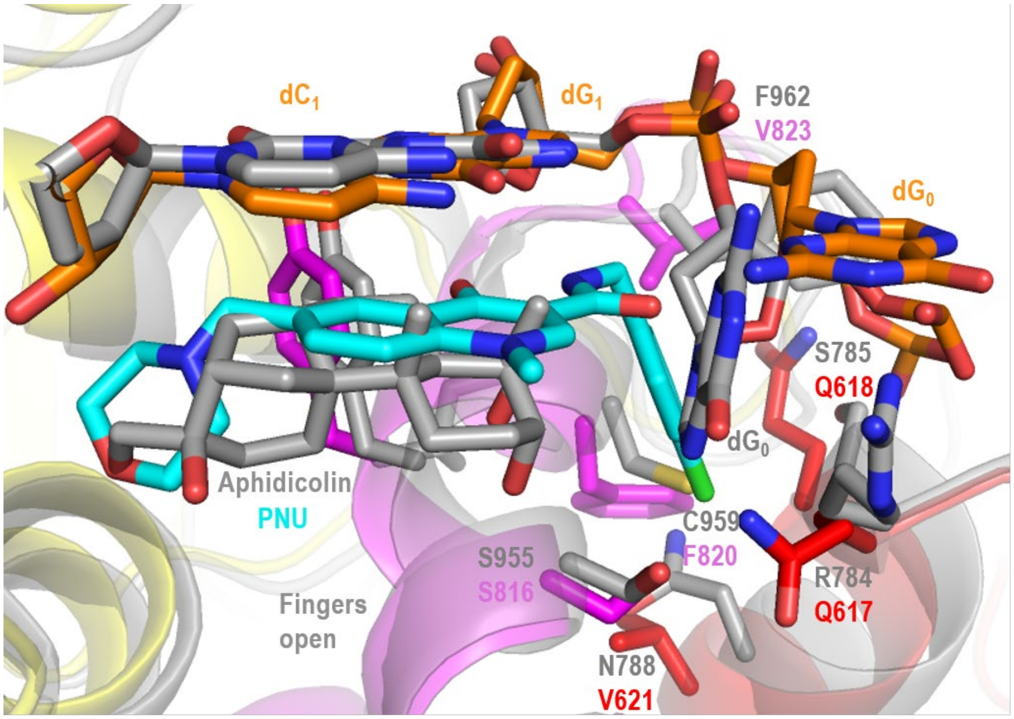

Supplementary Figure 10. Overlay of HSV1-pol with human pol α – aphidicolin ternary complex (PDB:4Q5V [http://dx.doi.org/10.2210/pdb4q5v/pdb]), R.M.S.D.= 2.3 Å. Pol α is shown as a grey cartoon with nucleic acid and aphidicolin shown as grey sticks.

Supplementary Table 1. Data collection and refinement statistics.

	HSV1 Pol Ternary Complex
Data collection	
Space group	$P4_1$
Cell dimensions	
a, b, c (Å)	181.3 181.3 233.7
α, β, γ (°)	90, 90, 90
Resolution (Å)	181.3-3.5 (3.6-3.5)
R_{merge}	0.178 (1.217)
I / σI	8.0 (1.8)
Completeness (%)	100.0 (100.0)
Redundancy	6.6 (7.2)
$CC_{1/2}$	0.997 (0.702)
Refinement	
Resolution (Å)	57.3-3.5
No. reflections	94,722
R_{work} / R_{free}	0.2258 / 0.2455
No. atoms	
Macromolecule	17451
Ligand	60
B-factors	
Macromolecule	109.4
Ligand	81.2
R.m.s. deviations	
Bond lengths (Å)	0.003
Bond angles (°)	0.631

*Values in parentheses are for highest-resolution shell.

Supplementary Table 2. DNA sequence of MA-HIS8-SUMO-PreScission-HSV-1 DNA Pol(43-1197) E370A-pBAC1 expression plasmid.

Sequence
GGAACGGCTCCGCCCACTATTAATGAAATTAAAAATTCCAATTTTAAAAAACGCAGCAAGAGAAACATTTGTATGAAAGAATGCGTAG<br>AAGGAAAGAAAAATGTCGTCGACATGCTGAACAACAAGATTAATATGCCTCCGTGTATAAAAAAAATATTGAACGATTTGAAAGAAAA<br>CAATGTACCGCGCGGCGGTATGTACAGGAAGAGGTTTATACTAAACTGTTACATTGCAAACGTGGTTTCGTGTGCCAAGTGTGAAAAC<br>CGATGTTTAATCAAGGCTCTGACGCATTTCTACAACCACGACTCCAAGTGTGTGGGTGAAGTCATGCATCTTTTAATCAAATCCCAAG<br>ATGTGTATAAACCACCAAACTGCCAAAAAATGAAAACTGTCGACAAGCTCTGTCCGTTTGCTGGCAACTGCAAGGGTCTCAATCCTAT<br>TTGTAATTATTGAATAATAAAACAATTATAAATGTCAAATTTGTTTTTTATTAACGATACAAACCAAACGCAACAAGAACATTTGTAG<br>TATTATCTATAATTGAAAACGCGTAGTTATAATCGCTGAGGTAATATTTAAAATCATTTTCAAATGATTCACAGTTAATTTGCGACAA<br>TATAATTTTATTTTCACATAAACTAGACGCCTTGTCGTCTTCTTCTTCGTATTCCTTCTCTTTTTCATTTTTCTCTTCATAAAAATTA<br>ACATAGTTATTATCGTATCCATATATGTATCTATCGTATAGAGTAAATTTTTTGTTGTCATAAATATATATGTCTTTTTTAATGGGGT<br>GTATAGTACCGCTGCGCATAGTTTTTCTGTAATTTACAACAGTGCTATTTTCTGGTAGTTCTTCGGAGTGTGTTGCTTTAATTATTAA<br>ATTTATATAATCAATGAATTTGGGATCGTCGGTTTTGTACAATATGTTGCCGGCATAGTACGCAGCTTCTTCTAGTTCAATTACACCA<br>TTTTTTAGCAGCACCGGATTAACATAACTTTCCAAAATGTTGTACGAACCGTTAAACAAAAACAGTTCACCTCCCTTTTCTATACTAT<br>TGTCTGCGAGCAGTTGTTTGTTGTTAAAAATAACAGCCATTGTAATGAGACGCACAAACTAATATCACAAACTGGAAATGTCTATCAA<br>TATATAGTTGCTGATCAGATCTGATCATGGAGATAATTAAAATGATAACCATCTCGCAAATAAATAAGTATTTTACTGTTTTCGTAAC<br>AGTTTTGTAATAAAAAAACCTATAAATATAGGATCCATAAATATGGCCCACCACCACCATCATCACCACCACGGCAGCGACTCCGAGG<br>TGAACCAGGAGGCTAAACCCGAGGTGAAGCCCGAGGTGAAACCCGAAACCCACATCAATCTGAAGGTGAGCGACGGCAGCAGCGAGAT<br>TTTTTTCAAAATTAAGAAAACTACCCCTCTCCGCCGCCTCATGGAGGCCTTTGCCAAACGTCAGGGCAAAGAAATGGACAGCCTCACC<br>TTCCTCTACGATGGTATCGAGATCCAGGCTGATCAGACCCCCGAGGACCTCGACATGGAAGACAACGATATTATCGAAGCCCACCGCG<br>AACAAATTGGCGGCCTGGAGGTGCTGTTCCAAGGCCCTAACTTCTATAACCCTTACCTCGCTCCCGTCGGCACCCAACAGAAGCCTAC<br>TGGTCCCACCCAGCGCCATACTTACTACTCCGAGTGCGACGAATTCCGCTTCATCGCCCCCCGCGTGCTCGACGAGGACGCTCCCCCT<br>GAGAAGCGCGCTGGTGTGCACGACGGTCACCTGAAACGCGCTCCCAAGGTCTATTGCGGCGGTGACGAACGTGATGTCCTGCGTGTGG<br>GTAGCGGCGGCTTTTGGCCCCGTCGTTCCCGTCTGTGGGGTGGTGTCGATCACGCCCCTGCTGGTTTTAACCCTACCGTGACTGTCTT<br>CCACGTCTATGACATCCTCGAGAACGTGGAACACGCTTACGGCATGCGTGCTGCCCAGTTTCACGCTCGCTTCATGGACGCCATTACC<br>CCCACCGGCACCGTCATTACTCTCCTCGGCCTGACTCCTGAGGGCCATCGTGTGGCTGTGCATGTGTACGGTACCCGCCAGTACTTCT<br>ATATGAACAAAGAAGAGGTGGATCGTCATCTGCAGTGCCGTGCCCCTCGCGACCTGTGTGAGCGTATGGCCGCCGCTCTCCGTGAATC<br>CCCTGGTGCCAGCTTCCGTGGTATCTCCGCTGATCACTTCGAGGCTGAGGTGGTGGAGCGCACTGACGTGTATTACTACGAGACCCGT<br>CCTGCTCTCTTCTACCGTGTGTACGTCCGCTCCGGTCGTGTCCTCTCCTACCTCTGCGATAACTTCTGCCCCGCTATCAAGAAGTACG<br>AGGGCGGTGTGGACGCTACCACCCGCTTCATTCTGGATAACCCTGGCTTTGTCACCTTCGGTTGGTACCGCCTGAAACCTGGTCGCAA<br>CAACACTCTCGCCCAGCCTCGCGCTCCTATGGCCTTCGGCACTAGCAGCGACGTGGAGTTCAACTGCACCGCTGACAATCTGGCTATT<br>GAAGGCGGTATGAGCGACCTGCCCGCCTACAAGCTGATGTGCTTCGACATTGCCTGCAAGGCCGGCGGCGAGGATGAACTGGCTTTCC<br>CCGTCGCCGGCCACCCTGAGGATCTGGTGATCCAAATCTCCTGTCTCCTGTACGATCTGAGCACCACCGCTCTGGAACACGTGCTGCT<br>GTTCTCCCTGGGCAGCTGCGATCTGCCTGAGTCCCACCTCAATGAACTGGCTGCTCGCGGTCTGCCTACCCCTGTGGTCCTGGAGTTT<br>GATTCCGAGTTCGAGATGCTCCTGGCTTTCATGACCCTGGTGAAGCAGTACGGCCCCGAGTTCGTGACCGGCTACAATATCATCAACT<br>TCGACTGGCCCTTCCTCCTGGCCAAGCTCACCGACATCTATAAAGTCCCCCTCGATGGTTATGGCCGCATGAACGGCCGCGGCGTGTT<br>CCGTGTGTGGGATATCGGTCAATCCCACTTCCAAAAACGTAGCAAGATCAAGGTGAACGGCATGGTGAACATCGACATGTACGGCATC<br>ATTACCGACAAGATCAAGCTGAGCAGCTACAAGCTGAACGCCGTCGCTGAAGCTGTGCTGAAGGACAAAAAGAAGGATCTGTCCTACC<br>GCGACATCCCTGCTTATTATGCTGCTGGCCCCGCCCAGCGTGGTGTGATTGGTGAGTACTGCATTCAGGACAGCCTCCTGGTGGGCCA<br>ACTGTTTTTCAAGTTCCTCCCCCATCTGGAGCTGAGCGCTGTGGCTCGCCTGGCCGGCATCAACATCACCCGCACTATCTACGACGGT<br>CAGCAAATCCGCGTGTTCACCTGCCTCCTCCGTCTCGCCGACCAGAAGGGCTTCATTCTGCCCGACACCCAAGGTCGTTTCCGTGGTG<br>CTGGCGGTGAAGCCCCCAAACGTCCTGCCGCTGCTCGTGAAGACGAAGAACGTCCCGAAGAGGAGGGTGAAGATGAGGATGAACGCGA<br>AGAAGGCGGCGGTGAGCGCGAACCTGAAGGCGCCCGTGAAACTGCCGGCCGTCACGTCGGCTATCAGGGTGCTCGCGTGCACGATCCC<br>ACTTCCGGCTTCCATGTCAACCCCGTGGTCGGCTTTGACTTCGCTAGCCTCTACCCTTCCATTATCCAGGCCCACAATCTGTGCTTTT<br>CCACCCTCTCCCTCCGCGCTGACGCTGTGGCTCATCTGGAGGCTGGTAAGGATTACCTCGAGATCGAGGTGGGCGGTCGCCGTCTGTT<br>CTTCGTGAAGGCCCATGTGCGCGAGTCCCTGCTCAGCATCCTGCTGCGCGATTGGCTCGCTATGCGTAAGCAGATTCGTAGCCGCATC<br>CCTCAGAGCTCCCCCGAAGAGGCTGTGCTGCTCGATAAGCAGCAGGCCGCCATTAAAGTGGTCTGCAACTCCGTGTACGGTTTCACTG<br>GTGTGCAACACGGTCTGCTGCCCTGCCTGCACGTCGCTGCCACCGTCACTACCATCGGTCGTGAAATGCTGCTCGCTACCCGTGAGTA<br>TGTCCACGCTCGCTGGGCCGCTTTCGAGCAGCTCCTCGCTGATTTTCCCGAGGCCGCCGATATGCGCGCTCCTGGCCCCTACTCCATG<br>CGTATCATCTATGGTGACACCGACAGCATCTTCGTCCTCTGCCGTGGTCTCACCGCTGCCGGCCTCACCGCTATGGGCGACAAAATGG<br>CTAGCCATATCAGCCGTGCCCTGTTCCTCCCCCCTATTAAGCTCGAATGCGAAAAGACTTTCACCAAGCTGCTGCTGATCGCTAAAAA<br>GAAGTACATCGGCGTCATCTACGGTGGTAAGATGCTCATCAAGGGCGTCGACCTGGTGCGCAAAAACAATTGCGCCTTCATCAACCGT<br>ACCAGCCGCGCTCTGGTCGACCTCCTGTTCTACGATGATACCGTCAGCGGCGCTGCTGCCGCTCTGGCCGAACGCCCTGCTGAAGAGT<br>GGCTGGCTCGCCCTCTGCCTGAGGGCCTGCAGGCTTTCGGCGCTGTCCTCGTCGACGCTCACCGTCGTATCACCGACCCCGAACGTGA<br>CATTCAGGACTTTGTGCTGACTGCCGAACTGTCCCGTCACCCTCGCGCTTACACTAACAAGCGTCTGGCTCATCTCACCGTCTACTAC<br>AAACTGATGGCCCGTCGTGCCCAAGTCCCCTCCATCAAGGACCGCATCCCTTACGTCATTGTCGCCCAGACCCGTGAAGTGGAAGAGA<br>CTGTGGCTCGCCTCGCTGCCCTCCGTGAACTGGATGCTGCTGCCCCTGGTGACGAACCCGCTCCTCCCGCCGCTCTCCCTTCCCCTGC<br>TAAGCGCCCCCGCGAAACCCCCTCCCATGCTGACCCTCCTGGTGGCGCCAGCAAGCCCCGTAAGCTGCTGGTGTCCGAACTCGCTGAG<br>GACCCCGCCTACGCTATCGCTCATGGCGTGGCCCTGAACACTGACTACTACTTCTCCCATCTGCTCGGCGCCGCCTGTGTGACCTTTA<br>AGGCCCTGTTCGGCAACAACGCCAAGATCACTGAGAGCCTCCTCAAACGCTTCATCCCCGAAGTCTGGCATTGATAAGCGGCCGCACT

```
CGAGCACCACCACCACCACCACTAACCTAGGTAGCTGAGCGCATGCAAGCTGATCCGGGTTATTAGTACATTTATTAAGCGCTAGATT
CTGTGCGTTGTTGATTTACAGACAATTGTTGTACGTATTTTAATAATTCATTAAATTTATAATCTTTAGGGTGGTATGTTAGAGCGAA
AATCAAATGATTTTCAGCGTCTTTATATCTGAATTTAAATATTAAATCCTCAATAGATTTGTAAAATAGGTTTCGATTAGTTTCAAAC
AAGGGTTGTTTTTCCGAACCGATGGCTGGACTATCTAATGGATTTTCGCTCAACGCCACAAAACTTGCCAAATCTTGTAGCAGCAATC
TAGCTTTGTCGATATTCGTTTGTGTTTTGTTTTGTAATAAAGGTTCGACGTCGTTCAAAATATTATGCGCTTTTGTATTTCTTTCATC
ACTGTCGTTAGTGTACAATTGACTCGACGTAAACACGTTAAATAGAGCTTGGACATATTTAACATCGGGCGTGTTAGCTTTATTAGGC
CGATTATCGTCGTCGTCCCAACCCTCGTCGTTAGAAGTTGCTTCCGAAGACGATTTTGCCATAGCCACACGACGCCTATTAATTGTGT
CGGCTAACACGTCCGCGATCAAATTTGTAGTTGAGCTTTTTGGAATTATTTCTGATTGCGGGCGTTTTTGGGCGGGTTTCAATCTAAC
TGTGCCCGATTTTAATTCAGACAACACGTTAGAAAGCGATGGTGCAGGCGGTGGTAACATTTCAGACGGCAAATCTACTAATGGCGGC
GGTGGTGGAGCTGATGATAAATCTACCATCGGTGGAGGCGCAGGCGGGGCTGGCGGCGGAGGCGGAGGCGGAGGTGGTGGCGGTGATG
CAGACGGCGGTTTAGGCTCAAATGTCTCTTTAGGCAACACAGTCGGCACCTCAACTATTGTACTGGTTTCGGGCGCCGTTTTTGGTTT
GACCGGTCTGAGACGAGTGCGATTTTTTTCGTTTCTAATAGCTTCCAACAATTGTTGTCTGTCGTCTAAAGGTGCAGCGGGTTGAGGT
TCCGTCGGCATTGGTGGAGCGGGCGGCAATTCAGACATCGATGGTGGTGGTGGTGGTGGAGGCGCTGGAATGTTAGGCACGGGAGAAG
GTGGTGGCGGCGGTGCCGCCGGTATAATTTGTTCTGGTTTAGTTTGTTCGCGCACGATTGTGGGCACCGGCGCAGGCGCCGCTGGCTG
CACAACGGAAGGTCGTCTGCTTCGAGGCAGCGCTTGGGGTGGTGGCAATTCAATATTATAATTGGAATACAAATCGTAAAAATCTGCT
ATAAGCATTGTAATTTCGCTATCGTTTACCGTGCCGATATTTAACAACCGCTCAATGTAAGCAATTGTATTGTAAAGAGATTGTCTCA
AGCTCGGATCGATCCCGCACGCCGATAACAAGCCTTTTCATTTTTACTACAGCATTGTAGTGGCGAGACACTTCGCTGTCGTCGAGGT
TTAAACGCTTCCTCGCTCACTGACTCGCTGCGCTCGGTCGTTCGGCTGCGGCGAGCGGTATCAGCTCACTCAAAGGCGGTAATACGGT
TATCCACAGAATCAGGGGATAACGCAGGAAAGAACATGTGAGCAAAAGGCCAGCAAAAGGCCAGGAACCGTAAAAAGGCCGCGTTGCT
GGCGTTTTTCCATAGGCTCCGCCCCCCTGACGAGCATCACAAAAATCGACGCTCAAGTCAGAGGTGGCGAAACCCGACAGGACTATAA
AGATACCAGGCGTTTCCCCCTGGAAGCTCCCTCGTGCGCTCTCCTGTTCCGACCCTGCCGCTTACCGGATACCTGTCCGCCTTTCTCC
CTTCGGGAAGCGTGGCGCTTTCTCATAGCTCACGCTGTAGGTATCTCAGTTCGGTGTAGGTCGTTCGCTCCAAGCTGGGCTGTGTGCA
CGAACCCCCCGTTCAGCCCGACCGCTGCGCCTTATCCGGTAACTATCGTCTTGAGTCCAACCCGGTAAGACACGACTTATCGCCACTG
GCAGCAGCCACTGGTAACAGGATTAGCAGAGCGAGGTATGTAGGCGGTGCTACAGAGTTCTTGAAGTGGTGGCCTAACTACGGCTACA
CTAGAAGGACAGTATTTGGTATCTGCGCTCTGCTGAAGCCAGTTACCTTCGGAAAAAGAGTTGGTAGCTCTTGATCCGGCAAACAAAC
CACCGCTGGTAGCGGTGGTTTTTTTGTTTGCAAGCAGCAGATTACGCGCAGAAAAAAAGGATCTCAAGAAGATCCTTTGATCTTTTCT
ACGGGGTCTGACGCTCAGTGGAACGAAAACTCACGTTAAGGGATTTTGGTCATGAGATTATCAAAAAGGATCTTCACCTAGATCCTTT
TAAATTAAAAATGAAGTTTTAAATCAATCTAAAGTATATATGAGTAAACTTGGTCTGACAGTTACCAATGCTTAATCAGTGAGGCACC
TATCTCAGCGATCTGTCTATTTCGTTCATCCATAGTTGCCTGACTCCCCGTCGTGTAGATAACTACGATACGGGAGGGCTTACCATCT
GGCCCCAGTGCTGCAATGATACCGCGAGACCCACGCTCACCGGCTCCAGATTTATCAGCAATAAACCAGCCAGCCGGAAGGGCCGAGC
GCAGAAGTGGTCCTGCAACTTTATCCGCCTCCATCCAGTCTATTAATTGTTGCCGGGAAGCTAGAGTAAGTAGTTCGCCAGTTAATAG
TTTGCGCAACGTTGTTGCCATTGCTACAGGCATCGTGGTGTCACGCTCGTCGTTTGGTATGGCTTCATTCAGCTCCGGTTCCCAACGA
TCAAGGCGAGTTACATGATCCCCCATGTTGTGCAAAAAAGCGGTTAGCTCCTTCGGTCCTCCGATCGTTGTCAGAAGTAAGTTGGCCG
CAGTGTTATCACTCATGGTTATGGCAGCACTGCATAATTCTCTTACTGTCATGCCATCCGTAAGATGCTTTTCTGTGACTGGTGAGTA
CTCAACCAAGTCATTCTGAGAATAGTGTATGCGGCGACCGAGTTGCTCTTGCCCGGCGTCAATACGGGATAATACCGCGCCACATAGC
AGAACTTTAAAAGTGCTCATCATTGGAAAACGTTCTTCGGGGCGAAAACTCTCAAGGATCTTACCGCTGTTGAGATCCAGTTCGATGT
AACCCACTCGTGCACCCAACTGATCTTCAGCATCTTTTACTTTCACCAGCGTTTCTGGGTGAGCAAAAACAGGAAGGCAAAATGCCGC
AAAAAAGGGAATAAGGGCGACACGGAAATGTTGAATACTCATACTCTTCCTTTTTCAATATTATTGAAGCATTTATCAGGGTTATTGT
CTCATGAGCGGATACATATTTGAATGTATTTAGAAAAATAAACAAATAGGGGTTCCGCGCACATTTCCCCGAAAAGTGCCACCTGACG
CGCCCTGTAGCGGCGCATTAAGCGCGGCGGGTGTGGTGGTTACGCGCAGCGTGACCGCTACACTTGCCAGCGCCCTAGCGCCCGCTCC
TTTCGCTTTCTTCCCTTCCTTTCTCGCCACGTTCGCCGGCTTTCCCCGTCAAGCTCTAAATCGGGGGCTCCCTTTAGGGTTCCGATTT
AGTGCTTTACGGCACCTCGACCCCAAAAAACTTGATTAGGGTGATGGTTCACGTAGTGGGCCATCGCCCTGATAGACGGTTTTTCGCC
CTTTGACGTTGGAGTCCACGTTCTTTAATAGTGGACTCTTGTTCCAAACTGGAACAACACTCAACCCTATCTCGGTCTATTCTTTTGA
TTTATAAGGGATTTTGCCGATTTCGGCCTATTGGTTAAAAAATGAGCTGATTTAACAAAAATTTAACGCGAATTTTAACAAAATATTA
ACGTTTACAATTTCCCATTCGCCATTCAGGCTGCGCAACTGTTGGGAAGGGCGATCGGTGCGGGCCTCTTCGCTATTACGCCA
```

Supplementary Table 3. DNA sequence of all primers and plasmids used in this study.

Experiment/name	Sequence
AlphaLISA activity assay/template	5’GAGGTCAAAACAGCGTGGATGGCGTCTCCAGGCGATCTGACGGTTCACTAAACGAGC
AlphaLISA activity assay/primer (DIG - digoxigenin)	5’DIG-AGCTCGTTTAGTGAACC
Crystallography/template	5’AATGGTAGGGGAAGGATCGTATGGCCT
Crystallography/primer	5’AGGCCATACGATCCTTCCCCTAC
